# A novel solid state photocatalyst for living radical polymerization under UV irradiation

**DOI:** 10.1038/srep20779

**Published:** 2016-02-11

**Authors:** Qiang Fu, Thomas G. McKenzie, Jing M. Ren, Shereen Tan, Eunhyung Nam, Greg G. Qiao

**Affiliations:** 1 Polymer Science Group, Department of Chemical and Biomolecular Engineering, The University of Melbourne, Parkville, Melbourne, VIC 3010, Australia

## Abstract

This study presents the development of a novel solid state photocatalyst for the photoinduced controlled radical polymerization of methacrylates under mild UV irradiation (*λ*_max_ ≈ 365 nm) in the absence of conventional photoinitiators, metal-catalysts or dye sensitizers. The photocatalyst design was based on our previous finding that organic amines can act in a synergistic photochemical reaction with thiocarbonylthio compounds to afford well controlled polymethacrylates under UV irradiation. Therefore, in the current contribution an amine-rich polymer was covalently grafted onto a solid substrate, thus creating a heterogeneous catalyst that would allow for facile removal, recovery and recyclability when employed for such photopolymerization reactions. Importantly, the polymethacrylates synthesized using the solid state photocatalyst (ssPC) show similarly excellent chemical and structural integrity as those catalysed by free amines. Moreover, the ssPC could be readily recovered and re-used, with multiple cycles of polymerization showing minimal effect on the integrity of the catalyst. Finally, the ssPC was employed in various photo-“click” reactions, permitting high yielding conjugations under photochemical control.

Since its discovery in 1998^1^, reversible addition−fragmentation chain transfer (RAFT) polymerization has become one of the most versatile controlled radical polymerization (CRP) techniques, as it is tolerant to a wide variety of reaction conditions and chemical functionalities, and can also be performed using conventional free-radical polymerization set-ups^2^,^3^,^4^,^5^,^6^,^7^. The generally accepted mechanism for a conventional RAFT polymerization relies on a crucial initiation step, where a radical is created from an added free radical initiator. The radicals derived from the initiator react with a thiocarbonylthio (TCT) compound (i.e. the RAFT agent) during the RAFT pre-equilibrium^8^. Extensive studies have been conducted to exert control over RAFT radical polymerizations through various physical and chemical stimuli that can activate certain radical initiators to begin the RAFT process. For example, Cai *et al*. achieved a well-controlled RAFT polymerization of methyl acrylate (MA) by choosing long-wavelength UV as an irradiation source and employing a suitable photoinitiator^3^. Kamigaito *et al*. have reported a photoinduced RAFT polymerization using a dinuclear manganese complex [Mn_2_(CO)_10_] initiator upon visible light irradiation^9^. By utilizing a novel azoinitiator with a half-life time of 10 h at 44 °C, Perrier *et al*. reported the rapid preparation of high-order multi-block copolymers with quantitative yields (>99% of each block)^10^.

Recently, there has been a dramatic resurgence of interest in photocontrolled radical polymerization (PRP) employing TCT compounds, which are stimulated by various different means to activate the TCT group and induce controlled polymerization processes^6^,^11^,^12^,^13^,^14^,^15^,^16^. Direct photoactivation of TCTs has been demonstrated by the groups of Bai^6^ and Johnson^17^, where the PRP of MA was mediated by a trithiocarbonate (TTC) under UV irradiation. During our previous studies, we discovered that direct photolysis of TCTs allowed for excellent control over acrylate and acrylamide monomers with both visible (blue light) and UV irradiation sources^14^. However, utilizing these systems for methacrylate monomers resulted in a significant loss of control due to possible degradation of the TCT, or more specifically the fragmented thiyl radical^2^,^6^.

Boyer *et al*. developed a powerful photochemically mediated RAFT polymerization by using various photoredox catalysts, whereby electron transfer to the TCT species was the key step prior to TCT fragmentation and the radical generation required to initiate polymerization^12^,^13^. This process, termed photoinduced electron transfer RAFT (i.e., PET-RAFT), has been shown to work for a wide range of photocatalysts including metal-complexes^12^, organic compounds^16^, porphyrin-derived structures^18^, and even isolated chlorophyll-A^19^. By taking advantage of a novel tertiary amine catalyst (TAC) and TCT two component system, we have reported on a synergistic PRP of methacrylates in the *absence* of conventional photoinitiators^20^. This system is proposed to act via a single electron photoreduction of the TCT in the presence of the TAC, avoiding the direct photolysis of the TCT and thus potentially increasing the stability of the fragmented species, resulting in a lower degradation and minimized side reactions. This was shown to dramatically improve the structural integrity of the formed polymethacrylates (i.e., molecular weight distribution, chain end fidelity, etc), compared to when the TCT was activated by direct UV photolysis. Additionally, the photoactivated nature of the polymerization imparts good temporal control, as demonstrated by “on-off” experiments whereby polymerization is effectively ceased in the absence of irradiation and resumed when the light source is turned back on.

Natural photosystems, particularly living cells that actively harvest solar energy, have inspired generations of researchers to design various devices and technologies that attempt to mimic natural photoelectrochemical processes^21^. In line with this we looked to photosynthesis, where photochemical reactions are mediated at the cell membrane in a heterogeneous surface reaction, and used it as the inspiration to transfer our tertiary amine (TA) catalyzed photopolymerization from a homogeneous to a heterogeneous system. This was proposed to have several significant benefits, most notably the ease with which the product can be purified and the catalyst recovered and re-used. Conversion of the TACs to the solid state was achieved in a straightforward manner by coating a solid substrate with a TA-rich polymer film, thus mimicking the thylakoid cell membrane found in plant leaves. The photocatalyst therefore consists of numerous TA units which can donate electrons to a TCT in solution upon UV irradiation, allowing for activation of the TA-catalyzed PRP.

In this study, we report on the development of a versatile solid state photocatalyst (ssPC) prepared by simply coating poly(2-dimethylamino)ethyl methacrylate (PDMAEMA) onto a solid substrate. The advantages of such a catalyst include low toxicity and facile removal/recovery post-polymerization in comparison with conventional soluble photoinitiators or photoactive complexes. The ultimate aim is to prepare a nature-inspired heterogeneous photocatalyst that can effectively catalyze a PRP process and provide convenience for post-polymerization purification.

## Results

### Design and fabrication of solid state photocatalyst

**Figure 1:**
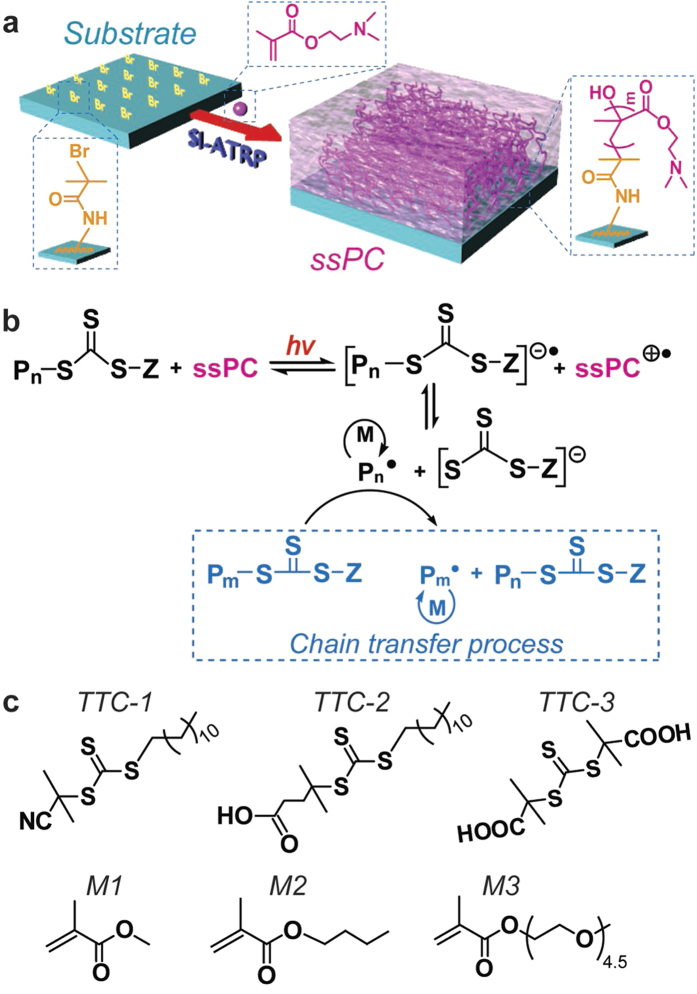
Schematic illustration for the preparation of solid state photocatalyst (ssPC). (**a**) The preparation of polymer coated solid state photocatalyst (ssPC) *via* SI-ATRP ([DMAEMA] = 200 mM, [DMAEMA]:[CuBr_2_]: [Me_6_TREN]:[Na-ascorbate] = 200:1:3:10). (**b**) The proposed mechanism of photocontrolled radical polymerization (PRP) using ssPC. (**c**) List of chemical structures of compounds investigated in this study: 2-cyano-2-propyl dodecyl trithiocarbonate (TTC-1), 4-cyano-4-(dodecyl-sulfanylthio-carbonyl)sulfanyl pentanoic acid (TTC-2), 2,2′-(thiocarbonyl-bis(sulfanediyl))-bis(2-methyl-propanoic acid) (TTC-3), methyl methacrylate (MMA, M1), *n*-butyl methacrylate (BMA, M2) and oligo(ethylene glycol) methyl ether methacrylate (OEGMA, M3).

Tertiary amines (TAs) are known to facilitate electron transfer in traditional photochemistry^22^. Therefore, the design of an ideal heterogeneous system with high activity and efficiency for photopolymerization reactions was to coat a TA-rich polymer onto a solid substrate to afford a solid state photocatalyst (ssPC) (Fig. 1a). Upon applying the ssPC in a photocontrolled radical polymerization (PRP) process, photoirradiation is able to induce an electron transfer from the TA-rich polymer chains to a trithiocarbonate (TTC), leading to the generation of a TTC radical anion ([P_n_-S-C(=S)-S-Z]^•−^) and amine radical cations ([NR_3_]^•+^) located on the ssPC surface. Subsequently, the TTC radical anion can reversibly cleave to generate a reactive carbon-centered radical (P_n_^•^) capable of initiating the polymerization of vinyl monomers, as well as a resonance-stabilized TTC anion ([S-C(=S)-S-Z]^−^) (Fig. 1b). The additional electron transfer process (from ssPC to TTC) is expected to protect the TTC from direct photolysis and thus improve the control observed in the PRP of methacrylate-type monomers. To examine our design, PRPs of methacrylates will be conducted in the presence of TTCs (Fig. 1c) and the ssPC under mild UV irradiation.

**Figure 2:**
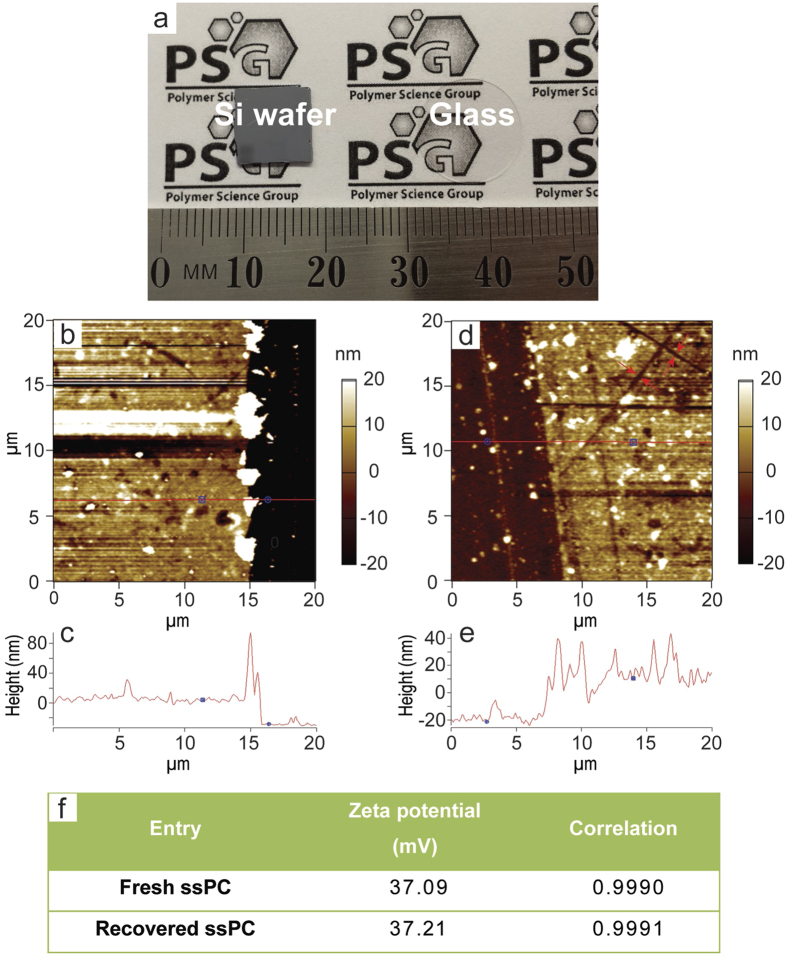
Characterization of the ssPCs. (**a**) Digital photo of the prepared ssPC based on silicon wafer (left) and glass cover slip (right) substrates. (**b**–**e**) 2D AFM images showing scratched zones of the freshly prepared PDMAEMA film before (**b**,**c**), and after (**d**,**e**) being used for PRP reactions. The z-profiles (**c**,**e**) are associated with the solid red lines across the scratches. (**f**) Zeta potentials of the freshly prepared ssPC and the recovered ssPC. Produced with permission for the PSG logo.

2-(Dimethylamino)-ethyl methacrylate (DMAEMA) was employed to generate the TA-rich polymer coating on various solid substrates. Firstly, 3-(2-bromoisobutylamido)-propyl-(triethoxy)-silane (BIBAPTES) was immobilized on the substrate (silicon wafer/glass cover slip) as the initiator for the subsequent surface-initiated atom transfer radical polymerization (SI-ATRP) process. Activators re-generated by electron transfer (ARGET) ATRP of DMAEMA was then conducted by immersing these substrates into an aqueous solution containing CuBr_2_, Me_6_TREN, sodium ascorbate and the monomer (DMAEMA). After 6 hours, the PDMAEMA-coated substrates were removed and washed with Milli-Q water to remove any unreacted monomer or catalyst residues. AFM, zeta potential (Fig. 2), XPS (Fig. S1 and S2) and TGA (Fig. S3) measurements were conducted to confirm efficient coating of the substrate with the polymeric film formed by the SI-ATRP process. A control experiment to monitor the molecular weight of grafted PMAEMA was also conducted by adding a non-surface bound ATRP initiator to the reaction mixture (Fig. S3).

### Photocontrolled radical polymerizations of methacrylates in the present of ssPC

**Figure 3:**
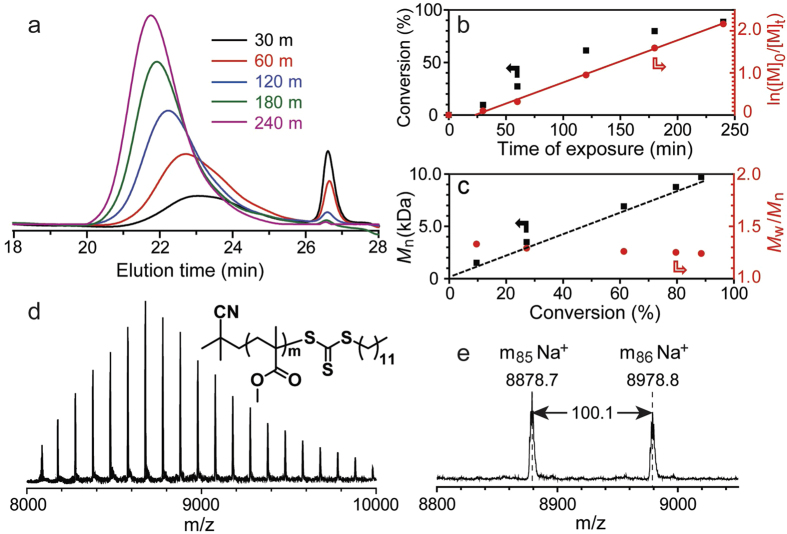
Characterization of PMMA prepared by PRP in the presence of the ssPC. (**a**) GPC evaluation, (**b**) kinetic study, (**c**) molecular weight/molecular weight distribution data (the dotted line represents the theoretical molecular weight based on ^1^H NMR analysis), and (**d**,**e**) MALDI-ToF-MS analysis.

PRP of a model monomer (methyl methacrylate (MMA)) was then conducted using various TTCs and the prepared ssPCs under mild UV irradiation (Fig. S4, broad emission, *λ*_max_ ∼ 365 nm). In general, deoxygenated mixtures containing MMA (100 equiv.) and TTC (1 equiv.) in DMSO (50%, v/v) with the ssPC were found to yield well-defined PMMA (*M*_n GPC_ = 9,700 Da, *M*_w_/*M*_n_ = 1.24, Fig. 3 and Table S1). The local concentration of the ssPC (*Local*_*TA@ssPC*_) was also calculated (Fig. S5 and Table S2) and compared to the concentration of free TA used in our previous report^20^.

Since the PDMAEMA coating is covalently attached to the substrate, the formed ssPCs are expected to present good chemical resistance and tolerance towards different solvents and/or monomers. Table 1 summarizes the characterization data of the prepared PMMA, poly(n-butyl methacrylate) (PBMA) and poly(oligo(ethylene glycol) methyl ether methacrylate) (POEGMA) using different TTCs and silicon/glass-based ssPCs (Fig. S6).

### Recyclability of the ssPC

**Figure 4:**
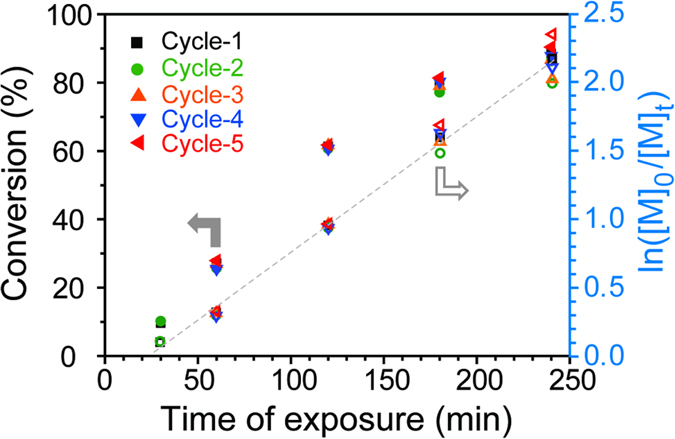
Recyclability of ssPC as demonstrated by PRP of MMA in the presence of ssPC. The kinetic study was conducted using freshly prepared ssPC (Cycle-1), and recovered ssPC (Cycle-2 to Cycle-5).

After a few PRP cycles, the recovered ssPC was characterized by zeta potential (Fig. 2f) and XPS measurements (Fig. S7 and S8). To examine the recyclability of the ssPC, PRP of MMA was also carried out in the presence of recovered ssPC under UV irradiation. After 4 hours of irradiation, PMMA was obtained with good control of the molecular weights and low molecular weight distribution (MWD) (Fig. S9 and Table S3). The kinetic study reveals the characteristics of a typical “living” polymerization, *i.e.* ln([M]_0_/[M]_t_) increases linearly with the increase of reaction time (Fig. 4).

### Temporal “ON-OFF” control over the polymerization process

**Figure 5:**
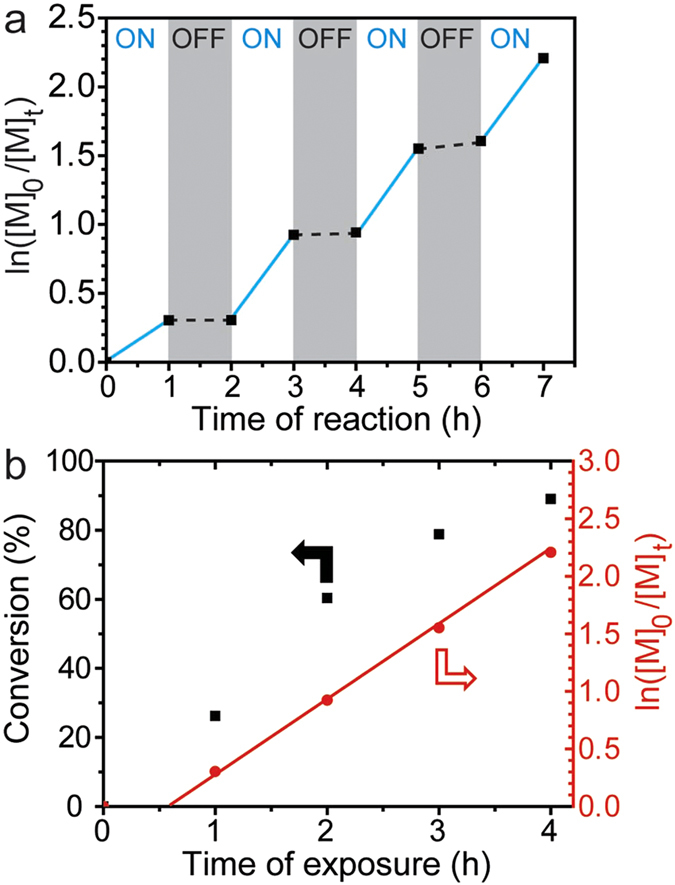
“ON-OFF” cycling of the PRP reaction to 365 nm UV light. Plots of PMMA polymerization: (**a**) ln([M]_0_/[M]_t_) *vs.* reaction time and (**b**) monomer conversion and ln([M]_0_/[M]_t_) *vs.* exposure time for the ssPC system.

Subsequently, to demonstrate temporally controlled PRP, the mixture of MMA, TTC1 and ssPC was exposed to an alternating light “ON” and “OFF” environment. In the absence of light no (or minimal) polymerization was observed. When the light was “ON”, the polymerization proceeded as expected (Fig. 5).

### Preparation of (*pseudo*)block copolymers

**Figure 6:**
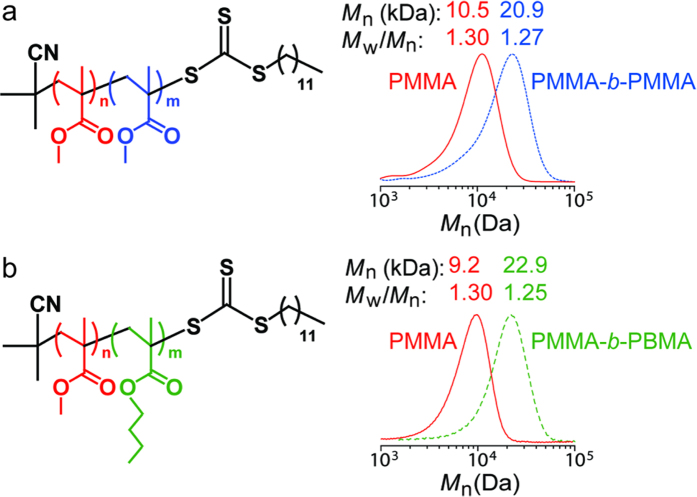
Chain extension experiments. GPC traces of (**a**) PMMA-*b*-PMMA and (**b**) PMMA-*b*-PBMA (*pseudo*)diblock copolymers using ssPC.

To further confirm the end-group fidelity of the polymer structure, chain extension experiments were performed to generate the (*pseudo*)block copolymers PMMA-*b*-PMMA (Fig. 6a) and PMMA-*b*-PBMA (Fig. 6b) using sequential PRPs.

### Photo-“click” reactions

**Figure 7:**
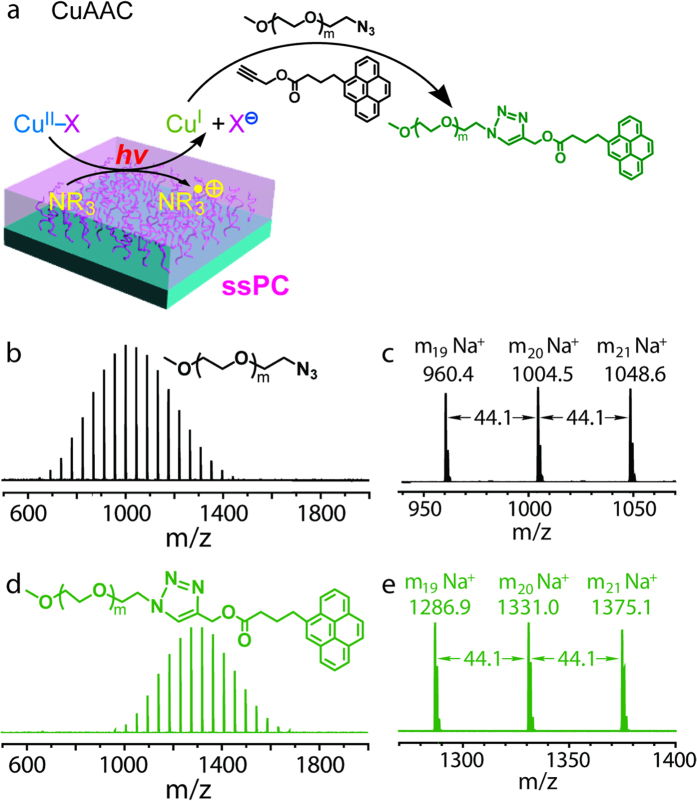
The preparation of pyrene functionalized PEG (PEG-Py) *via* ‘CuAAC’ reaction using Cu^II^ and ssPC. (**a**) Schematic illustration of the ‘CuAAC’ reaction using ssPC. MALDI-TOF mass spectrometry results of (**b**,**c**) azide functionalized PEG (PEG-azide) and (**d**,**e**) the resultant pyrene functionalized PEG (PEG-Py).

Finally, we investigated the possibility of applying the ssPC in a copper-catalyzed azide-alkyne cycloaddition (CuAAC) reaction. A modal ‘click’ reaction of an azide functionlized poly(ethylene glycol) (PEG-azide) and propargyl 1-pyrene butyrate (PPy) was conducted in the presence of Cu^II^ and the ssPC (Fig. 7).

## Discussion

The ssPCs formed are shown in Fig. 2a, with surface areas of ca. 1 cm^2^ for silicon wafer and ca. 1.77 cm^2^ for glass cover slip. Figure 2b,c illustrates the thickness profiles for the freshly prepared PDMAEMA film on a silicon wafer. Generally, the polymer film displays a thickness of *ca.* 40 nm as determined by AFM scratch analysis. The formed PDMAEMA film also shows a zeta potential value of 37.09 mV (Fig. 2f), which is in good agreement with previous report^23^. The deconvoluted C 1s, O 1s, Br 3d and N 1s spectra shown in Fig. S2a-d provide detailed information about surface functional groups present on the functionalized silicon wafer. After the SI-ATRP process, the C 1s, O 1s, Br 3d and N 1s signals have been altered as revealed in Fig. S2e-h. For example, the deconvoluted C 1s spectrum signal at 289.0 eV became stronger, which is attributed to the carbons in the C=O bond in the methacrylate moieties of PDMAEMA (Fig. S2e). The broader O 1s signal of the PDMAEMA coating (Fig. S2f) can be differentiated into 2 peaks at 532.1 eV and 534.2 eV, which are ascribed to the oxygen on the substrate (Si-O) and the carbonyl group (C=O) of the methacrylates. The disappearance of Br 3d signal of PDMAEMA coating is attributed to the hydrolysis of bromine post-polymerization (Fig. S2g). The replacement of the terminal –Br group by –OH in aqueous ATRP or SET-LRP has been previously reported in detail^24^. In addition, the N 1s spectrum also presented a stronger signal at 399.0 eV (Fig. S2h), indicating the TA became rich on the photocatalyst surface. The formed PDMAEMA brushes on the ssPC (0.3 cm^2^, 22.9512 mg) have a mass of ~0.02 mg (~1.25 × 10^−4^ mmol) as determined by TGA (Fig. S3a). All of these results therefore demonstrate the successful preparation of a TA-rich PDMAEMA film on a solid substrate. In order to investigate the molecular weight of the grafted PDMAEMA, we further conducted a control experiment by adding an ATRP initiator in the reaction mixture. Under the same reaction conditions, the PDMAEMA formed in solution was collected and characterized by GPC (*M*_n_ = 38.4 kDa, *M*_w_/*M*_n_ = 1.24, Fig. S3b).

Kinetic investigations revealed high conversion of monomer (~90%) within 4 hours following a short initial induction period (Fig. 3b). Following this induction period, a linear dependence of ln([M]_0_/[M]_t_) with time demonstrated the rate of the polymerization to be first order with respect to monomer conversion, while *M*_n NMR_ (calculated based on ^1^H NMR data in Table S1) increased linearly with time and the molecular weight distribution (MWD) remained narrow (*M*_w_/*M*_n_ ~ 1.3) throughout the reaction (Fig. 3c). In addition, using the described system the synthesized PMMA displayed good retention of α and ω end-groups at high monomer conversions, as evidenced by MALDI-ToF MS (Fig. 3d,e).

Based on the simulation results shown in Table S2 and Fig. S5, the average diameter of DMAEMA is determined as 0.689 nm. Thus the amount of tertiary amine on each ssPC is estimated to be 4.0 × 10^−4^ mmol/cm^2^ (with a thickness of 40 nm) by Eq. S2. The TGA measurement also reveals a similar amount of tertiary amine (*ca.* 4.2 × 10^−4^ mmol/cm^2^, 0.02 mg weight loss for a ssPC with an area of 0.3 cm^2^) compared with the calculated number. In our previous study, we generally applied ~94 mmol of tertiary amine for a PRP (i.e. [Me_6_TREN] = 23 mM) with the same [monomer]/[TTC]^20^. Interestingly, the catalytic activity and efficiency of the presented ssPC are slightly higher than that of the free amine, whereby ~80% MMA conversion was achieved after 4 hours of irradiation. This is suggestively attributed to the higher local concentration (~100 mM, determined by Eq. S3) of TA immobilized on the ssPC, leading to a faster electron transfer process in the homogeneous PRP system.

As observed previously^20^, in the absence of a suitable source of tertiary amines (*i.e.* the ssPC in this case) polymerization still occurs upon UV irradiation, however the product is of high molecular weight (*M*_n GPC_ = 42.1 kDa) with a broad MWD (*M*_w_/*M*_n_ = 1.88), indicating that the polymerization is not well controlled (Entry 1 in Table 1 and Fig. S6a). Both hydrophobic (Entries 2–7) and hydrophilic (Entry 8) polymethacrylates with low MWD (*M*_w_/*M*_n_ ~ 1.3) at high monomer conversions (>90%) were obtained when utilizing the ssPC, indicating good controllability over the PRP process (Fig. S6b–h). This is attributed to the formation of amine radical cations on the ssPC, and TTC radical anions in solution, providing a beneficial pathway compared with direct photolysis (Fig. 1b). This result is in agreement with previous reports at high monomer conversion for methacrylate monomers^2^. The resultant PMMAs using Si or glass-based substrates were identical (Entries 2 and 3, respectively), revealing negligible effect of the substrate on the PRP process. The PRP of MMA was also carried out using a higher [M]/[TTC] ratio of 500 (Entry 5 and Fig. S6e). The product is of high molecular weight (*M*_n GPC_ = 45.1 kDa) with a narrow MWD (*M*_w_/*M*_n_ = 1.33), demonstrating the good control of the present PRP for the synthesis of high molecular weight polymers. The GPC chromatogram of POEGMA (Fig. S6h) shows an asymmetric curve and a small tail toward the low molecular weight region. This phenomenon is known to be due to the strong interaction between poly(ethylene glycol) chains and the chromatography column (THF as mobile phase)^25^. Therefore, we can conclude that the ssPC system presents high tolerance towards to different TTCs, monomers, solvents and substrates.

One unique feature of heterogeneous catalysts is their facile removal and recovery post-reaction. As shown in Fig. 2d,e, after a few PRP cycles, the polymer coating maintained similar thickness (*ca.* 40 nm) in comparison with the freshly prepared film. Aside from a few scratches, which may be attributed to damage from the stirring bar, no change in the ssPC could be observed visually. The recovered ssPC also has a zeta potential of 37.21 mV (Fig. 2f), which is nearly identical to that of the freshly prepared ssPC. This is also supported by the XPS measurements, where the recovered ssPC presented similar XPS spectrum (Fig. S7) and high resolution C 1s, O 1s and Br 3d spectra (Fig. S8) compared to the freshly prepared ssPC. This indicates the high chemical stability of the polymer coating and infers that the ssPC should retain its catalytic activity for subsequent PRPs. When the ssPC was used for multiple cycles, all recorded reaction kinetics showed characteristics of a typical “living” polymerization: namely, first-order kinetics with regards to monomer conversion (Fig. 4), and molecular weights increasing as a function of monomer conversion (Fig. S9 and Table S3). Despite the small deviation (± 2.05 *conv.* %), the overall activity is identical between the first use (Cycle-1) and all subsequent cycles. These results again demonstrate excellent recyclability and catalytic activity of the recovered ssPC.

The lack of any reaction in the dark suggested that the polymerization could be activated/deactivated by UV light, leading to controlled regulation of the polymerization process externally. To investigate this responsive nature of the PRP using a ssPC, the reactions were exposed to 365 nm UV light, reaching ~26% conversion within 1 hour. After removal of the light source, nearly complete cessation was observed over a further 1 hour period; re-exposure with UV light led to resumed polymerization. This cycle could be repeated several times up to high conversions (~90%), indicating efficient activation and deactivation of the polymerization process with light (Fig. 5a). Significantly, a linear increase in molecular weight versus conversion is obtained even with multiple “ON-OFF” light switching cycles, and the observation of first order kinetics through the course of the reaction demonstrated a controlled polymerization process (Fig. 5b). These data indicate that when light is removed from the system the TTC is oxidized to the stable and dormant state and upon re-exposure to light, in the presence of the ssPC, the TTC is able to efficiently and reversibly generate propagating radicals.

To further probe the “living” nature of this system, as well as provide additional evidence for the presence of active groups at the chain end, (*pseudo*)block copolymers were prepared. For example, irradiation of a mixture of MMA and TTC-1 in the presence of the ssPC afforded a well-defined PMMA derivative, with controlled molecular weight and low MWD. Without any purification, the resultant PMMA was utilized as a macro-TTC for *in situ* chain extension experiments by adding another 100 equiv. MMA, leading to well-defined PMMA-*b*-PMMA *pseudo-*block copolymers via a one-pot strategy. ^1^H NMR analysis also indicates that high conversions (>95%) are achieved in the preparation of starting PMMAs as well as the chain extension step (Table S4 and Fig. S10). The GPC profiles of the chain-extended products (blue trace) clearly shows a shift to a higher molecular weight region with little tailing in the homopolymer region (Fig. 6a). A diblock copolymer was also prepared using an isolated PMMA (conv. **~** 84%, Table S5) as macro-TTC in the PRP of BMA (Fig. 6b). GPC and ^1^H NMR (Fig. S11) analysis prove the successful preparation of PMMA-*b*-PBMA. In this case, the GPC profile of the chain-extended product (green trace) clearly shows a shift to a higher molecular weight region with no tailing observed. In addition, a *pseudo*-triblock copolymer (PMA-*b*-PMA-*b*-PMA) was synthesized *via* an *in situ* chain extension strategy (Table S6). The GPC trace of the resultant *pseudo*-triblock presents a monomodal distribution with a narrow MWD of 1.14 (Fig. S12). As revealed in our previous study^20^, TA is capable of catalysing the PRPs of various monomers, such as acrylates, styrene and acrylamide. Thus we can deduce that the ssPC is able to catalyse the PRP of a library of functional polymers.

A model photo-“click” reaction was then conducted to verify the proposed photoinduced electron transfer mechanism. As shown in Fig. 7a, the ssPC is able to reduce Cu^II^ to Cu^I^ through a photoredox process upon UV irradiation. The generated Cu^I^ is capable of catalysing the coupling reaction of PEG-azide and PPy. MALDI-ToF MS analysis of the resulting product shows a single monomodal series and a clear shift to higher molecular weights, indicating a quantitative yield of the coupling product (Fig. 7b,e). Additionally, the photo-‘click’ reaction of PEG-azide and an alkyne-functionalied poly(ɛ-caprolactone) (PCL-alkyne) was performed under same conditions. The GPC trace of the resulting block copolymer (Fig. S13a) shows a clear shift to the higher molecular weight region, indicating a high yield of the coupling product. ^1^H NMR analysis also reveals the formation of the characteristic triazole ring (Fig. S13b). These results provide strong evidence for the proposed photoinduced electron transfer mechanism.

In summary, we have reported a versatile polymer-coated solid state photocatalyst for the PRP of methacrylates. The ssPC presents advantages over conventional photoinitiators, metal/organic catalysts and dye sensitizers, as it can be facilely removed post-polymerization and easily recovered by simple purification methods. Another distinguishing feature of this ssPC is that it is easy to fabricate, inexpensive, relatively nontoxic, easy to handle, and amenable to a variety of functional groups, monomers and solvents. The polymers synthesized *via* this ssPC system show narrow molecular weight distributions and high end-group fidelity. The accessibility of the end-groups was confirmed by chain extension experiments to yield (*pseudo*)block copolymers of high purity. Negligible polymerization in the absence of light confers temporal control of the PRP process. In addition, this system is capable of efficiently catalysing CuAAC reactions in quantitative yield. Further studies investigating the scope for this ssPC system are in progress.

## Methods

### Preparation of the solid state photocatalyst (ssPC)

The procedure for the surface initiation (SI) from a silicon wafer has been described elsewhere^26^. A Si wafer (ca. 1 cm × 1 cm) or glass cover slip (diameter = 1.5 cm) was immersed overnight in a vial containing an initiator solution (5% BIBAPTES, 5% Milli-Q water and 90% absolute ethanol) at room temperature. The bromide functionalized Si wafer (or glass cover slip) was thoroughly washed with ethanol and Milli-Q water, and dried in vacuo. The surface initiated polymerization was conducted by Activators Regenerated by Electron Transfer (ARGET)-ATRP in aqueous system at room temperature^27^. All substrates functionalized with a bromo-initiator were placed in an air-tight container (containing DMAEMA (200 mM), CuBr_2_ (1 mM), Me_6_TREN (1 mM) and NaAsc (3 mM) in Milli Q water). After the specified reaction time at room temperature, the polymer-coated substrates were taken out, washed with Milli-Q water and dried in vacuo. The preparation of PDMAEMA films on the Si wafer (or glass cover slip) was confirmed by AFM and XPS measurements. Thereafter the substrates were further cut to smaller pieces (AREA = 0.2–0.5 cm^2^) for use.

### General procedure for PRP using ssPC

A solid state photocatalyst, freshly rinsed with THF, was put in a Schlenk tube charged with monomer (MMA: 0.94 g, 1.0 mL, 9.4 mmol), TTC (TTC-1, 34.5 mg, 0.094 mmol) DMSO (1 mL, 50 vol % w.r.t monomer), [MMA]:[TTC] = 100:1. The reaction mixture was degassed by 2 freeze-pump-thaw cycles then back-filled with argon. The UV light source was then switched “on”, and the reaction mixture was stirred under positive argon pressure. Samples were taken at timed intervals *via* a degassed syringe and immediately diluted with either CDCl_3_ or THF, for NMR and GPC analysis, respectively. MMA conversion was estimated from ^1^H NMR by integrating the peaks corresponding to the polymer methyl group (*δ*_H_ = 3.42 ppm, m, 3H, -OC***H***_***3***_) and the protons corresponding to the unsaturated methyacrylate double bond (*δ*_H_ = 5.35–6.0 ppm, m, 2H, ***H***_***2***_C=C-). The former peak accounts for all protons derived from the monomer species, while the latter is only unreacted monomer. Hence, the percentage of unreacted monomer can be calculated. The ssPC was recycled, washed with THF and dried by blowing argon for repeated experiments.

### “ON-OFF” reactions

The “ON-OFF” reactions were set up in the same fashion, however at a given reaction time the UV light source was turned off and the flask was sealed under argon, covered completely in aluminium foil and placed into a home-made ‘dark box’ for a designated time period. The polymerization was re-activated by irradiating the reaction mixture with UV again. Samples were taken at timed intervals via a degassed syringe and immediately diluted with either CDCl_3_ or THF, for NMR and GPC analysis, respectively.

### General procedure for chain extension experiments

(1) *In situ* chain extension experiments for preparation of PMMA-*b*-PMMA. After 4 hours a 1:2 (v/v) mixture of degassed MMA (100 equiv.) used for *pseudo*-block copolymerization and DMSO was added to the reaction mixture *via* a degassed syringe. Samples were taken after another 4 hours and measured using ^1^H NMR and GPC.

(2) *In situ* chain extension experiments for preparation of PMA-*b*-PMA*-b*-PMA. The *pseudo*-triblock copolymer was prepared via the same *in situ* strategy as described above. Samples characterized by ^1^H NMR and GPC.

(3) Chain extension experiments for preparation of PMMA-*b*-PBMA. After 3.5 hours polymerization, PMMA samples were taken via a degassed syringe and immediately diluted with either CDCl_3_ or THF, for NMR and GPC analysis, respectively. The resultant macro-TTCs (PMMA homopolymers) were isolated by precipitation in cold methanol, collected via filtration, and then dried under vacuum. Thereafter, a Schlenk tube was charged with the second monomer (BMA, 100 equiv.), macro-TTC (1 equiv.), DMSO (400 vol % w.r.t monomer) and ssPC. The reaction mixture was degassed by 2 freeze-pump-thaw cycles then back-filled with argon. The UV light source was then switched “on”, indicating the start of the chain extension reaction. Samples were taken after another 4 hours and characterized using ^1^H NMR and GPC analysis.

### Copper-catalyzed azide-alkyne cycloaddition (CuAAC) chemistry

(1) Coupling reaction of PEG and propargyl 1-pyrene butyrate. A 25 mL flask was charged with PEG-N_3_ (*M*_n_ = 1 kDa, 0.1 g, 0.1 mmol), propargyl 1-pyrene butyrate (PPy, 32.8 mg, 0.1 mmol), CuBr_2_ (22.3 mg, 0.1 mmol), DMSO (1 mL) and the ssPC. This was degassed by 2 freeze-pump-thaw cycles then back-filled with argon before the UV light source was switched on. The reaction was stopped after 24 h and immediately precipitated into cold DEE. After removal of DEE, the coupling product PEG-PPy was dried in vacuo (1 mbar) at 30 °C prior to MALDI-ToF MS analysis.

(2) Preparation of PEG-*b*-PCL copolymer via click chemistry. A 25 mL flask was charged with PEG-N_3_ (*M*_n_ = 1 kDa, 0.1 g, 0.1 mmol), *α*-alkyne PCL (*M*_n_ = 3.8 kDa, 0.38 g, 0.1 mmol), CuBr_2_ (22.3 mg, 0.1 mmol), DMSO (4.8 mL) and the ssPC. This was degassed by 2 freeze-pump-thaw cycles before the UV light source was switched on. The reaction was performed under argon positive pressure. The reaction was stopped after 24 h and immediately precipitated into cold methanol. After removal of methanol, the resultant PEG-*b*-PCL was dried in vacuo (1 mbar) at 30 °C prior to GPC and ^1^H NMR analysis.

### Gel-Permeation Chromatography (GPC)

GPC with THF as mobile phase was conducted using a Shimadzu system fitted with a Wyatt DAWN DSP multi-angle laser light scattering detector (690 nm, 30 mW) and a Wyatt OPTILAB EOS interferometric refractometer (690 nm). Three Agilent PLgel columns (MIXED-C; 5 μm bead size) were employed, operating at 1 mL per minute at a column temperature of 45 °C. To process the GPC data, the program ‘Astra’ by Wyatt technologies was used. All samples were filtered through 0.45 μm nylon filters prior to injection. When aqueous-phase GPC was employed, a separate Shimadzu liquid chromatography system was utilized, equipped with a Shimadzu RID-10 refractometer (λ = 633 nm), using three Waters Ultrahydrogel columns in series ((i) 250 Å porosity, 6 μm diameter bead size; (ii) and (iii) linear, 10 μm diameter bead size), operating at room temperature. The eluent was Milli-Q water containing 20% v/v acetonitrile and 0.1% w/v TFA at a flow rate of 0.5 mL·min^**−**1^. The molecular weight of the analyte was determined using a calibration based on narrow molecular weight distribution poly(ethylene glycol) standards.

### Nuclear Magnetic Resonance (NMR) Spectroscopy

^1^H NMR spectroscopy and ^13^C NMR spectroscopy were conducted on a Varian Unity 400 MHz spectrometer operating at 400 MHz, using the solvent deuterated chloroform (CDCl_3_) (Cambridge Isotope Laboratories) as reference and sample concentrations of approximately 10 mg·mL^**−**1^.

### Matrix-assisted laser desorption/ionization time of flight (MALDI-ToF) mass spectroscopy

MALDI-ToF MS was performed on a Bruker Autoflex III Mass Spectrometer operating in positive linear mode; the analyte, matrix (DCTB) and cationisation agent (NaTFA) were dissolved in THF at a concentration of 10 mg·mL^**−**1^, and then mixed in a volume ratio of 1:10:1. Then 0.3 μL of this solution was spotted onto a ground steel target plate and the solvent was allowed to evaporate prior to analysis. FlexAnalysis (Bruker) was used to analyze the data.

### X-ray photoelectron spectroscopy (XPS)

XPS analysis was performed on a VG ESCALAB 220i-XL spectrometer under ultra-high vacuum (6 **×** 10^**−**9^ mbar) to reveal the surface composition of the polymer coating. A fixed photon energy (Al Kα 1486.6 eV) was used. A survey scan was performed between 0 and 1200 eV with a resolution of 1.0 eV and pass energy of 100 eV. High resolution scans for C1s (276 to 296 eV) and O1s (522 to 542 eV) were also conducted with a resolution of 0.05 eV and a pass energy of 20 eV.

### Atomic force microscopy (AFM)

AFM images of air-dried PDMAEMA films on silicon wafers were acquired with a JPK NanoWizard2 Bio-AFM or a MFP-3D Asylum Research instrument. Typical scans were conducted in intermittent contact mode with silicon cantilevers (NSC/CSC) (MicroMesh, Bulgaria). Image processing and surface roughness analysis were performed using JPK image processing software and Nanoscope software programs (Bruker), respectively. Film thicknesses were estimated by film scratching (mechanical removal) and by tracing a profile along the film and the scratched zone. The thickness measurements reported represent mean values over 3 different analysis sites per substrate. Physical analysis of scratched films by AFM showed good agreement with ellipsometry data.

### Zete potential

Zeta potential measurements were conducted using a flat plate streaming potential apparatus described elsewhere^28^ at a temperature of 25 °C. Streaming potentials were recorded for flow in both directions and the zeta potential was calculated using the Helmholtz-Smoluchowski equation.

### Thermogravimetric analysis (TGA)

TGA was performed on a PerkinElmer Pyris-1 thermogravimetric analyzer, and the samples were heated from 30 to 600 °C at a heating rate of 10 K min^−1^ under an atmosphere flow (20 mL min^−1^).

## Additional Information

**How to cite this article**: Fu, Q. *et al*. A novel solid state photocatalyst for living radical polymerization under UV irradiation. *Sci. Rep.* **6**, 20779; doi: 10.1038/srep20779 (2016).

## Supplementary Material

Supplementary Information

## Figures and Tables

**Table 1:** Summary of GPC and ^1^H NMR data of the polymers synthesized using ssPCs.

Entry^*a*^	Solvent	TTC	Monomer	Ratio [TTC]:[M]	ssPCs	Conv.^*c*^ (%)	*M*_n, th_^*c*^ (Da)	*M*_n, GPC_^*d*^ (Da)	*M*_w_/*M*_n_^*d*^
1	DMSO	TTC-1	MMA	1:100	N/A	>99	10,400	42,100	1.88
2	DMSO	TTC-1	MMA	1:100	Si	90	9,400	10,300	1.24
3	DMSO	TTC-1	MMA	1:100	Glass	92	9,600	10,400	1.25
4	DMSO	TTC-1	BMA	1:100	Si	91	13,300	14,500	1.29
5	DMSO	TTC-1	MMA	1:500	Si	85	43,300	45,100	1.33
6	DMSO	TTC-2	MMA	1:100	Si	91	9,600	10,300	1.25
7	DMSO	TTC-2	BMA	1:100	Si	90	13,200	14,300	1.28
8	H_2_O/Dioxane^*b*^	TTC-3	OEGMA	1:200	Si	>99	59,700	55,600	1.35

^*a*^The reactions were performed in solvents for 4h at room temperature using UV lamp (*λ*_max_ = 365 nm) as light source. ^*b*^H_2_O/Dioxane = 50/50 v/v%. ^*c*^Monomer conversion and theoretical molecular weight determined by ^1^H NMR spectroscopic analysis. ^*d*^Molecular weight and molecular weight distribution determined by GPC.
