# Supplementary material for: A novel solid state photocatalyst for living radical polymerization under UV irradiation
Source: Sci Rep. 2016 Feb 11;6:20779. doi: 10.1038/srep20779 (PMC4749958; doi:10.1038/srep20779)
Supplement: Supplementary Information [file srep20779-s1.pdf]

Title: A novel solid state photocatalyst for living radical polymerization under UV irradiation

Authors: Qiang Fu[1], Thomas G. McKenzie[1], Jing M. Ren[1], Shereen Tan[1], Eunhyung Nam[1] & Greg G. Qiao[1]

[1]Polymer Science Group, Department of Chemical and Biomolecular Engineering, The University of Melbourne, Parkville, Melbourne, VIC 3010, Australia.

Correspondence and requests for materials should be addressed to G.G.Q.
(Email: gregghq@unimelb.edu.au)

1. Experimental section

Materials. (3-Aminopropyl)-triethoxysilane (APTES, ≥ 99 %), 2-bromoisobutyryl bromide (BIBB, 98 %), tris(2-aminoethyl)amine (96 %), formic acid (98 %), formaldehyde (25 %), ammonium hydroxide (NH_4OH, 28-30 %), sulfuric acid (H_2SO_4, 97 %), 1-dodecanethiol (> 98 %), benzyl bromide (anhydrous, 99.8%), hydrochloric acid (HCl, 37 %), Aliquat® 336, carbon disulfide (CS_2, anhydrous, ≥ 99%), 2-cyano-2-propyl dodecyl trithiocarbonate (TTC-1, 97 %), 4-cyano-4-((dodecylsulfanylthiocarbonyl)sulfanyl) pentanoic acid (TTC-2, 97%), trans-2-(3-(4-*tert*-butylphenyl)-2-methyl-2-propenylidene) malon- nitrile (DCTB, 98 %), sodium trifluoroacetate (NaTFA, > 99 %), 3-mercaptopropionic acid (> 99 %), *p*-toluenesulfonic acid monohydrate (PTSA, > 98 %), trifluoroacetic acid (TFA, 99 %), 1-pyrenebutyric acid (97 %), propargyl alcohol (99 %) and sodium ascorbate (NaAsc, ≥ 98 %) were purchased from Sigma-Aldrich and used as received. Monomers 2-(dimethylamino)-ethyl methacrylate (DMAEMA, 98 %), methyl acrylate (MA, 99 %, Aldrich), methyl methacrylate (MMA, 99 %, Aldrich), *n*-butyl methacrylate (BMA, 99 %, Aldrich) and oligo(ethylene glycol) methyl ether methacrylate (OEGMA, M_n = 300 Da, Aldrich) were passed over basic alumina to remove inhibitors prior to use. Copper(II) bromide ($CuBr_2$, 99 %) was obtained from Ajax chemicals. Deuterated chloroform ($CDCl_3$, 99.9 %) was purchased from Cambridge Isotope Laboratories, Inc. Triethylamine (TEA) was distilled over calcium hydride under argon. Tetrahydrofuran (THF) was distilled from benzophenone and sodium metal under argon. AR grade potassium hydroxide (KOH), anhydrous magnesium sulphate ($MgSO_4$), hydrogen peroxide (H_2O_2, 30 %), isopropanol (IPA), methanol (MeOH), formic acid, dichloromethane (DCM), chloroform, acetone, n-hexane, 1,4-dioxane, diethyl ether (DEE) and other solvents were purchased from Chem-Supply Pty. Ltd. and used without further purification. High-purity water with a resistivity higher than 18 MΩ·cm was obtained from an in-line Millipore Rios/Origin water purification system. Silicon wafers were obtained from MMRC Pty. Ltd. and cut to approximately 1 cm × 1 cm pieces, followed by cleaning with Piranha solution (H_2SO_4/H_2O_2 = 7/3). After 30 min, the pieces were rinsed with Milli-Q water and sonicated in IPA and water (1/1 v/v) solution for 30 min. The pieces were washed thoroughly with Milli-Q water and thermally treated at 60 °C in RCA solution (Milli-Q water/H_2O_2/NH_4OH = 5/1/1) for 30 min. Finally, the pieces were rinsed thoroughly with Milli-Q water and stored in IPA before use. The glass cover slips (Livingstone Int. Pty. Ltd.) were rinsed thoroughly with Milli-Q water and stored in IPA before use. Tris(2-(dimethylamino)ethyl)amine (Me_6TREN), 3-(2-bromoisobutyramido)propyl(triethoxy)-silane (BIBAPTES), 2,2'-(thiocarbonyl-bis(sulfanediyl))-bis(2-methyl-propanoic acid) (TTC-3), propargyl 1-pyrenebutyrate (PPy), *propargyl*-terminated *poly(ε-caprolactone) (PCL-alkyne,* $M_{n\ GPC}$ = 4.0 kDa*)* and *α*-azido PEG (PEG-azide, $M_{n\ GPC}$ = 1.1 kDa) were all synthesised in our laboratory[20-22] and well characterized, the details for which are available in the Supporting Information. UV light source used for all experiments was a commercial nail-curing lamp ('Beaufly-nail lamp', 220V) fitted with 4 × 9W bulbs with λ_{max} ~ 365 nm.

Synthesis of tris(2-(dimethylamino)ethyl)amine (Me_6TREN). Tris(2-(dimethylamino)ethyl)amine (Me_6TREN) was prepared according to the literature[1]**.** Tris(2-aminoethyl amine) (12 mL, 80 mmol) was placed in a round-bottom flask containing HCl in MeOH (100 mL, 3 M). After reaction at room temperature for 1 hour, the solid residue was collected by filtration. The solid was washed with MeOH and dried in vacuo. 18 g of the solid was dissolved in a mixture of H_2O, formic acid and formaldehyde (1 : 6 : 6, volume ratio). The reactant was stirred at 120 °C until CO_2 release had stopped. The resulting solution was dissolved in 10 % NaOH solution (200 mL). The oily layer was extracted in DEE, followed by evaporation of DEE. Me_6TREN was collected from vacuum distillation at 70 °C as colorless oil. ^{1}H NMR (400 MHz, $CDCl_3$, δ_H ppm): 2.58-2.52 (t, 2H,

$(CH_3)_2NC\mathit{H_2}CH_2$-), 2.34-2.30 (t, 2H, $(CH_3)_2NCH_2C\mathit{H_2}$-), 2.18-2.14 (s, 6H, $(C\mathit{H_3})_2N$-). ^{13}C NMR (400 MHz, $CDCl_3$, δ_C ppm): 57.5, 53.0, 45.9.

Synthesis of 3-(2-bromoisobutyramido)propyl(triethoxy)-silane (BIBAPTES). 3-(2-Bromoisobutyramido)propyl(triethoxy)-silane (BIBAPTES) was synthesized according to literature[2]. The 3-aminopropyltriethoxysilane (10 g, 45.2 mmol) and triethylamine (10.1 g, 100 mmol) were placed in a round-bottom flask containing 100 mL of anhydrous THF. 2-bromoisobutyryl bromide (16.1 g, 70 mmol, 1.55 eqv.) was dissolved in 30 mL of anhydrous THF and the solution was added dropwise to the mixture of APTES and TEA placed in an ice bath over 60 min under vigorous stirring. The solution was allowed to warm to room temperature, and stirred overnight. The precipitate of triethylamine bromide (TEA HBr) was removed by filtration. The solution was then concentrated in vacuo (1 mbar) and product was re-dissolved in 200 mL of DCM. The organic solution was neutralized by washing with 0.1 M HCl solution, saturated $NaHCO_3$ solution and H_2O. Then the resulting solution was stirred overnight with anhydrous $MgSO_4$, filtered and concentrated in vacuo (1 mbar) at 30 ^oC. The product BIBAPTES with dark brown color was obtained. ^{1}H NMR (400 MHz, $CDCl_3$, δ_H ppm): 3.80-3.72 (m, 2H, $CH_3C\boldsymbol{H_2}O$-), 3.40-3.25 (m, 2H, -$C\boldsymbol{H_2}NH$-), 1.93-1.91 (s, 6H, -$C(C\boldsymbol{H_3})_2$-Br), 1.64-1.61 (m, 4H, -$CH_2C\boldsymbol{H_2}CH_2$-), 1.24-1.21 (t, 3H, $C\boldsymbol{H_3}CH_2O$-), 0.62-0.58 (t, 2H, -$SiC\boldsymbol{H_2}$-). ^{13}C NMR (400 MHz, $CDCl_3$, δ_C ppm): 172, 62.0, 53.4, 42.7, 32.1, 22.8, 13.8, 6.3.

Synthesis of 2,2'-(thiocarbonyl-bis(sulfanediyl))-bis(2-methylpropanoic acid) (TTC-3). CTA-3 was synthesized via a procedure reported in the literature[3]. Carbon disulfide (5.378 g, 70.64 mmol), chloroform (21.558 g, 0.180 mol), acetone (9.536 g, 0.180 mol), and Aliquat® 336 (1.101 g, 2.72 mmol) were dissolved in hexane (50 mL) in a 250 mL round bottom flask under a nitrogen atmosphere. The flask was placed in an ice/water bath. After the solution was cooled in an ice water bath for 20 min, a 50% sodium hydroxide aqueous solution (18.540 g, 0.231mol) was added dropwise into the flask. The mixture turned a deep yellow color after ~ 15 min and then a brownish red color after 1 h. The reaction was allowed to proceed at 0 ℃ overnight. 170 Deionized water (100 mL) was added into the flask, followed by dropwise addition of a concentrated HCl solution (30 mL) until the solution was acidic. The organic layer was separated and bubbled with nitrogen to remove the volatiles. The resultant slurry was filtered by vacuum filtration, and the collected solid was stirred in toluene/hexanes (v/v: 50/50). 2,2'-(thiocarbonylbis(sulfanediyl))bis(2-methylpropanoic acid) was obtained as a yellow powder. ^{1}H NMR (400 MHz, $CDCl_3$, δ_H ppm): 1.67 (m, 12H, -CH_3). ^{13}C NMR (400 MHz, $CDCl_3$, δ_C ppm): 180.0, 55.8, 25.14.

Synthesis of propargyl 1-pyrenebutyrate (PPy). 1-Pyrenebutyric acid (2.88 g, 10 mmol, 1.0 equiv) was dissolved in chloroform (50 mL) in a round bottomed flask under nitrogen and cooled to 0 ℃. Oxalyl chloride (1.03 mL, 12 mmol, 1.2 equiv) was then added slowly into the flask. Two drops of dimethyl formamide (DMF) were added as a catalyst for the reaction. The reaction mixture was stirred at room temperature for 20 h with constant stirring. The solvent and any excess oxalyl chloride was removed by rotary evaporation to obtain the acid chloride. The acid chloride was directly used in the next step without further purification. The acid chloride (1.0 equiv.) was dissolved in chloroform (20 mL) and added slowly to a solution of propargyl alcohol (0.87 mL, 15 mmol, 1.5 equiv) dissolved in chloroform (50 mL) and under nitrogen. The mixture was then stirred for 3 h. The chloroform and excess propargyl alcohol were removed in vacuo. The crude product was purified by flash chromatography (elution: hexane/DCM = 1/1 to DCM/MeOH = 95/5) to yield the propargyl 1-pyrenebutyrate. Rf (hexane/DCM = 1/1): 0.45; ^{1}H-NMR (400 MHz, $CDCl_3$, δ_H ppm): 8.31-7.85 (m, 9H, ArH), 4.71 (d, 2H, -$OCH_2C{\equiv}CH$), 3.43-3.39 (m, 2H, -$PyCH_2$-), 2.54-2.48 (m, 3H, -$CH_2C(O)$- and -$C{\equiv}CH$), 2.26-2.17 (m, 2H, -CH_2-); ^{13}C NMR (400 MHz, $CDCl_3$, δ_C

ppm): 172.7, 135.6, 131.6, 131.1, 130.2, 128.9, 127.6, 127.6, 127.5, 126.9, 126.0, 125.3, 125.1, 125.1, 125.0, 124.9, 123.4, 75.0, 52.0, 51.7, 33.7, 32.8, 26.8.

Synthesis of *α*-azido PEG. The mono-azido-functionalized PEG (PEG-azide) was synthesized via a procedure reported in the literature[4]. PEG monomethyl ether (M_n = 1 kDa, 2 g, 2 mmol, 1 equiv) was initially dried via azeotropic distillation with toluene (20 mL). Subsequently, triethylamine (2.4 mmol, 1.2 equiv) and dichloromethane (20 mL) were added and the mixture was cooled to 0 ^{o}C before methanesulfonyl chloride (3 mmol, 1.5 equiv) was added dropwise. The reaction was kept at 0 ^{o}C for 30 min and then at room temperature for 12 h. The reaction was filtered and the filtrate was concentrated in vacuo. The residue was dissolved in DMF (20 mL), and NaN_3 (20 mmol, 10 equiv) was added. The reaction mixture was heated to 65 ^{o}C for 12 h, cooled to room temperature and concentrated in vacuo. The residue was dissolved in water (50 mL) and then washed with dichloromethane (25 mL × 2). The organic extracts were collected, washed with water (50 mL) and saturated NaCl (50 mL × 2), dried ($MgSO_4$), filtered, and concentrated in vacuo to afford the desired PEG-N_3. ^{1}H NMR (400 MHz, $CDCl_3$, δ_H ppm): 3.85-3.45 (m, 4H, -CH_2CH_2O-) ppm, 3.41-3.37 (s, 3H, -OCH_3 and t, 2H, -CH_2N_3). MALDI-ToF MS: M_n = 1.07 kDa, PDI = 1.02.

Synthesis of azide-functionalized PCL *via* ROP. A typical procedure to synthesize azide-functionalized PCL (PCL-azide) was reported in the literature[5]. Propargyl alcohol (56 mg, 1 mmol, 1 equiv.) and CL (4.57 g, 40 mmol, 40 equiv.) were dissolved in 50 mL DCM in a dried 100 mL flask under argon. Then to the reaction mixture, MSA in anhydrous DCM (6 mL, 0.5 mol L^{-1}, 3 equiv.) was injected under argon atmosphere *via* syringe. The reaction was stirred at 25 ^{o}C for 4 h and the crude product were concentrated and precipitated into cold methanol (400 mL). The precipitate was isolated by filtration and dried *in vacuo* at 40 ^{o}C for 24 h. ($[M]_0/[I]_0$ = 50, conversion = 80.0 % *via* GC-MS); $M_{n\ GPC}$ = 4.0 kDa, M_w/M_n = 1.19; $M_{n\ NMR}$ = 3.7 kDa. ^{1}H NMR (400 MHz, $CDCl_3$, δ_H ppm): 4.66 (m, 2H, CH≡CC$\boldsymbol{H_2}$-), 4.12-4.02 (m, 2H, -C$\boldsymbol{H_2}$O-), 3.70-3.62 (t, J = 6.4 Hz, 2H, -C$\boldsymbol{H_2}$OH), 2.46 (m, 1H, C$\boldsymbol{H}$≡C-), 2.35-2.24 (m, 2H, -C(=O)C$\boldsymbol{H_2}$-), 1.72-1.56 (m, 4H, -C$\boldsymbol{H_2}$$CH_2C\boldsymbol{H_2}$-), 1.42-1.33 (m, 2H, -$CH_2C\boldsymbol{H_2}$$CH_2$-).

2. Preparation of the solid state photocatalyst (ssPC)

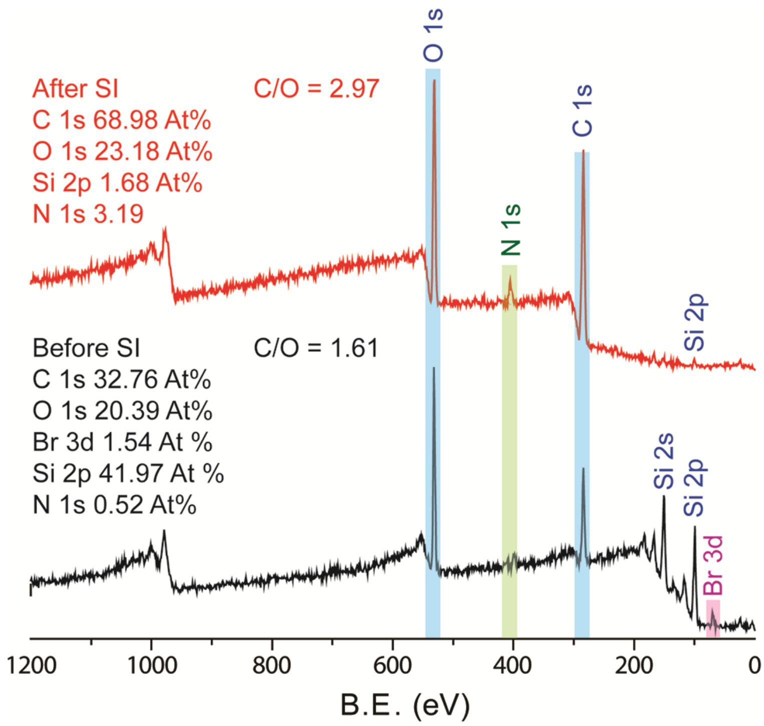

Figure S1. XPS wide scan spectra of the ATRP-initiator modified silicon wafer (before SI-ATRP) and the prepared PDMAEMA coat (after SI-ATRP).

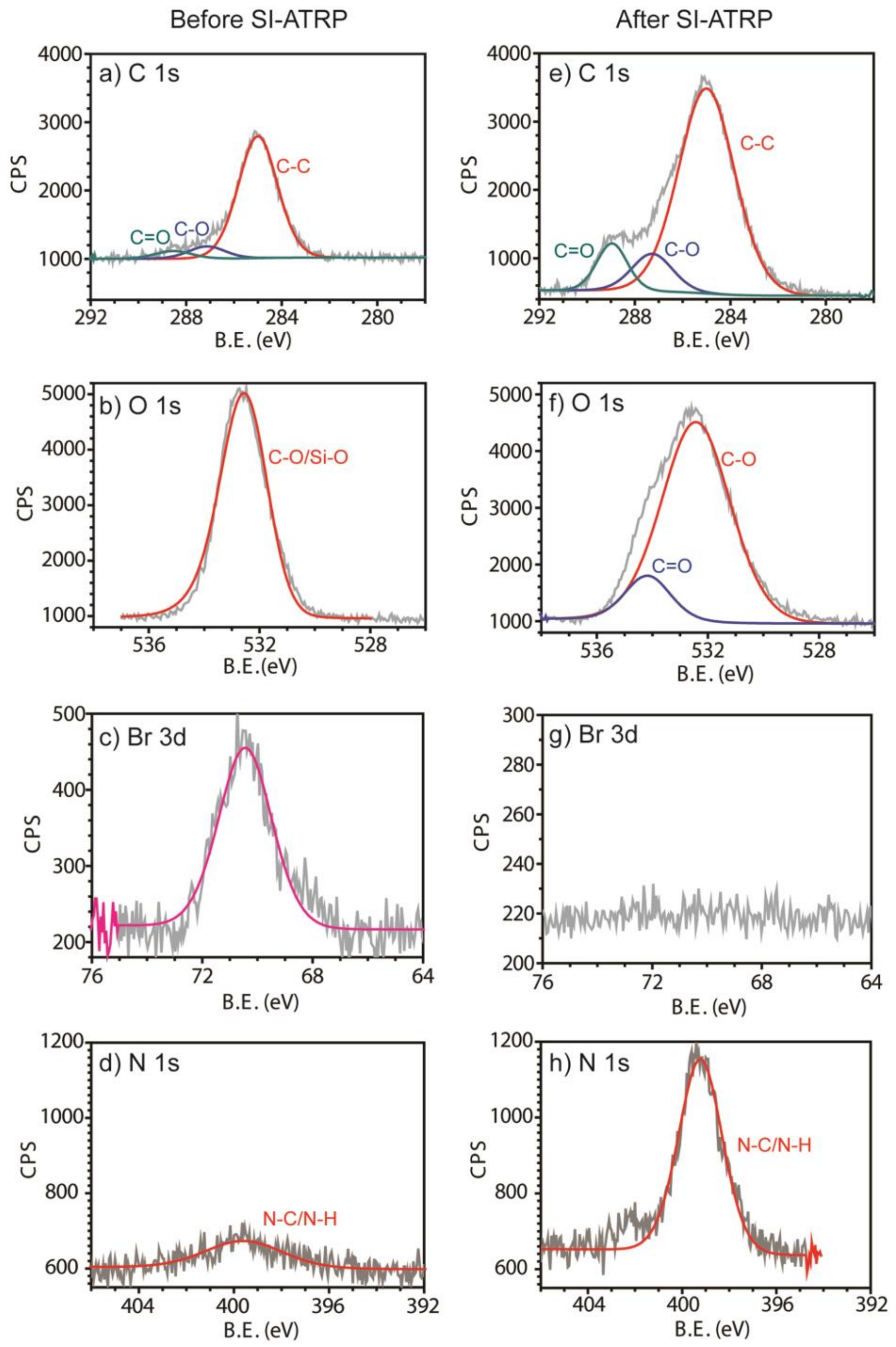

Figure S2. High-resolution XPS a) C 1s, b) O 1s, c) Br 3d and d) N 1s spectra of the ATRP-initiator functionalized Si wafer (before SI-ATRP); and e) C 1s, f) O 1s, g) Br 3d and h) N 1s spectra of the performed ssPC (after SI-ATRP), respectively.

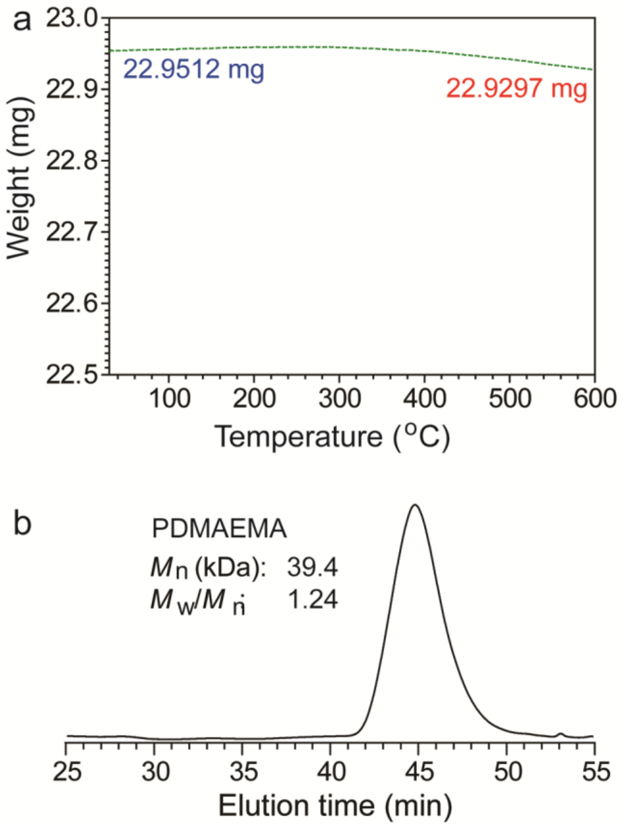

Figure S3. (a) TGA trace for the freshly prepared ssPC and (b) the GPC profile of PDMAEMA prepared by ARGET-ATRP.

3. Light Source

UV source used for all experiments was a commercial nail-curing lamp ('Beaufly-nail lamp', 220V, fitted with 4 × 9W bulbs with λ_{max} ~ 365 nm).

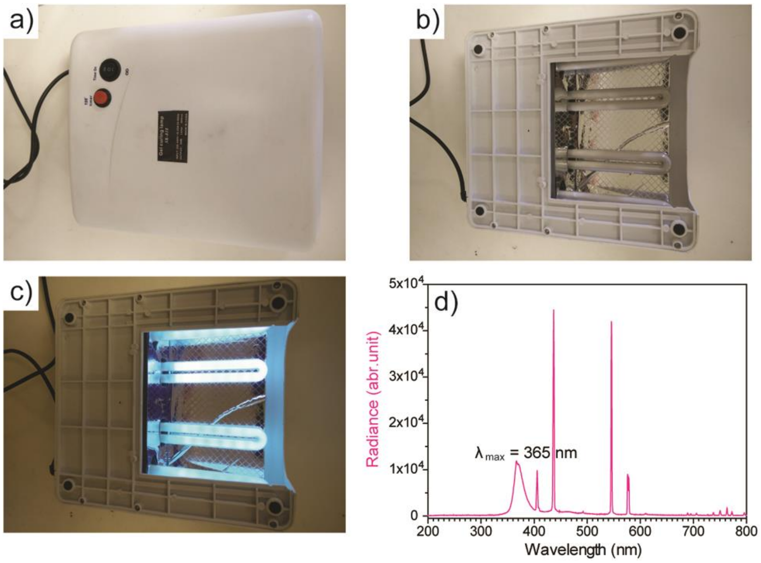

Figure S4. (a-c) Digital images of the "Off-On" UV lamp. (d) Emission spectrum of the UV lamp.

4. Kinetic study of poly(methyl methacrylate) for Figure 4

In this experiment, a 25 mL flask was charged with monomer (MMA: 1.78 mL, 16.7 mmol), TTC-1 (58 mg, 0.167 mmol), solid state photocatalyst (Si wafer 0.5 cm^2) and DMSO (50 vol % w.r.t monomer), [MMA]:[TTC] = 100:1. After polymerization, the MMA conversion was estimated from ^1H NMR by integrating the peaks corresponding to methyl group at δ_H = 3.5-3.7 ppm, (s, 3H, -COOC$\boldsymbol{H_3}$) and the protons corresponding to the unsaturated methacrylate double bond (δ_H = 5.3-6.0 ppm, m, 3H, C$\boldsymbol{H_2}$=C(CH_3)-). These peaks account for all protons derived from the monomer species, from which the percentage of remaining unreacted monomer can be calculated. The theoretical molecular weight was calculated using the equation:

$$M_{n,th} = [M]_0/[CTA]_0 \times \%\ \text{conv.} \times M_{r,\,M} + M_{r,\,TTC} \qquad \text{(S1)}$$

where $[M]_0$ is the initial concentration of monomer, $[CTA]_0$ is initial chain transfer agent concentration, % conv. is the monomer conversion estimated from ^1H NMR, $M_{r,\,M}$ and $M_{r,\,TTC}$ are the molecular weights of monomer and TTC, respectively.

Table S1. PRP of MMA as a function of polymerization time[a].

Entry	Polymerization time (min)	Conv.[b] (%)	$M_{n,th}$[b] (kDa)	$M_{n,\,GPC}$[c] (kDa)	M_w/M_n[c]
1	30	9.6	1.31	1.51	1.33
2	60	27.2	3.07	3.49	1.29
3	120	61.3	6.49	6.90	1.26
4	180	79.7	8.32	8.75	1.25
5	240	88.5	9.21	9.70	1.24

[a] Supplementary data to Fig. 3. [b] Determined by ^1H NMR. [c] Deterrmined by THF GPC.

5. Local concentration of ssPC

Table S2. Relating molecule size and molecular weight[a]

Molecular weight (Da)	Diameter (nm)
**1,000,000**	13
**100,000**	6.2
**10,000**	2.9
**1,000**	1.3
**100**	0.62

[a] z-Average diameters obtained for various known molecular weight polystyrene samples dissolved in toluene. (Data source: Malvern website http://www.malvern.com)

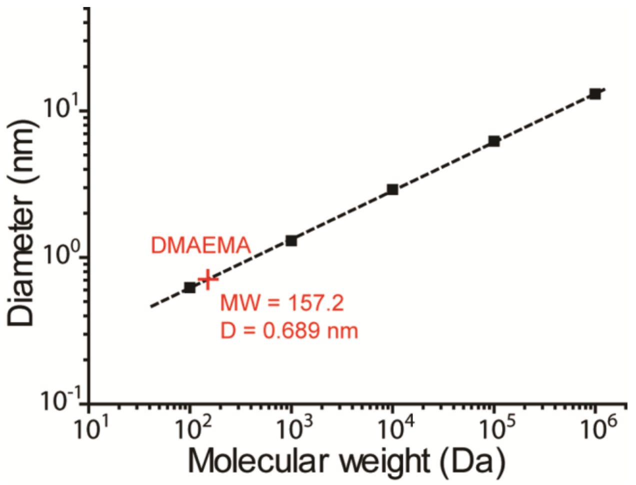

Figure S5. Plot of the log z-average diameter vs. log molecular weight for polystyrene in toluene.

For example, a ssPC has the PDMAEMA coating (Area = 1 cm^2, thickness = 40 nm), and the amount of TA on the ssPC can be estimated using the equation below:

$$n_{TA@ssPC} = \left(\frac{V_{PDMAEMA}}{V_{DMAEMA} \times N_A} \right) \approx 4 \times 10^{-4} mmol \qquad \text{(S2)}$$

Where $n_{TA@ssPC}$ is the amount of tertiary amine on the ssPC (Area = 1 cm^2, thickness = 40 nm), N_A is the Avogadro constant of 6.02×10^{23}, $V_{PDMAEMA}$ is the volume of PDMAEMA coating on the ssPC and V_{DMAEMA} is the dimension of DMAEMA.

Thus, the local concentration of the ssPC ($Local_{TA@ssPC}$) is calculated as follow:

$$[Local_{TA@ssPC}] = \left(\frac{n_{TA@PCD}}{V_{PDMAEMA}} \right) \approx 100mM \qquad \text{(S3)}$$

6. Supplementary data to Table 1

In this study, a 25 mL flask was charged with monomer, TTC, ssPC and solvent (50 vol % w.r.t monomer), [M]:[TTC] = 100:1. This was de-gassed *via* two freeze-pump-thaw cycles before the UV light source was switched on. The reaction was performed under argon positive pressure. Samples were taken after 6 h and immediately diluted with either $CDCl_3$ or THF, for NMR and GPC analysis, respectively.

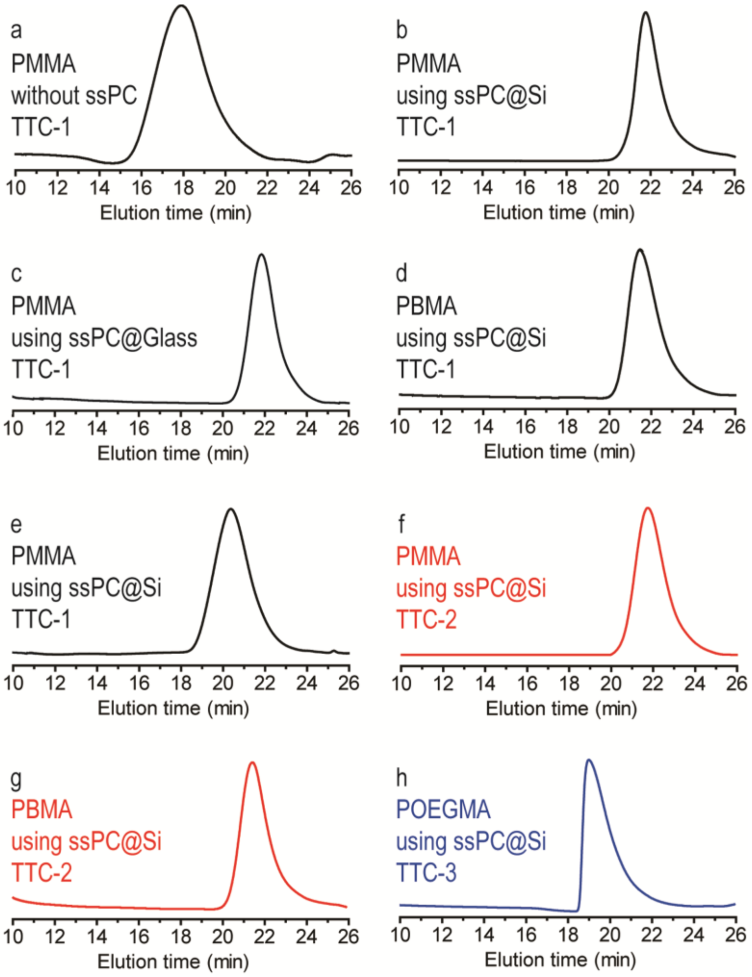

Figure S6. GPC traces of polymethacrylayes obtained from PRP using ssPCs (a-h correspond to Entry 1-8 in Table 1).

7. Characterization on the recovered ssPC

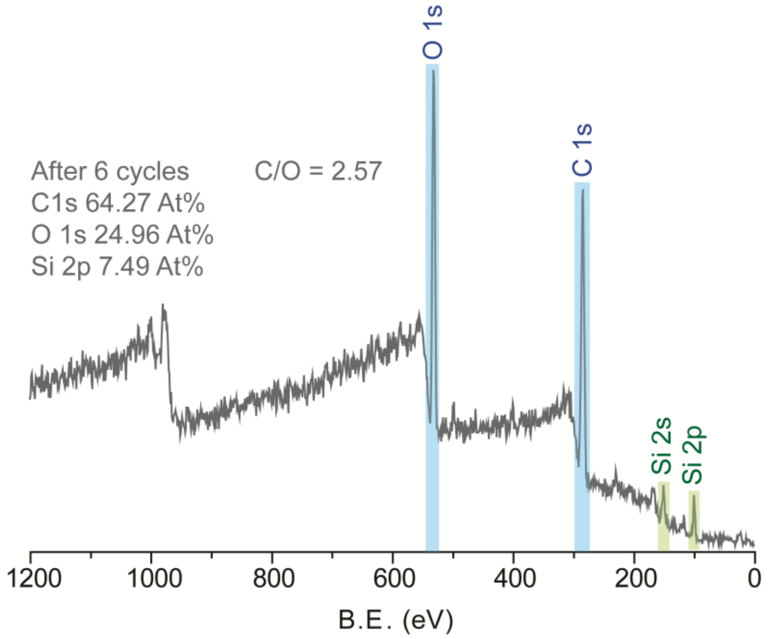

Figure S7. XPS wide scan spectrum of the recovered ssPC.

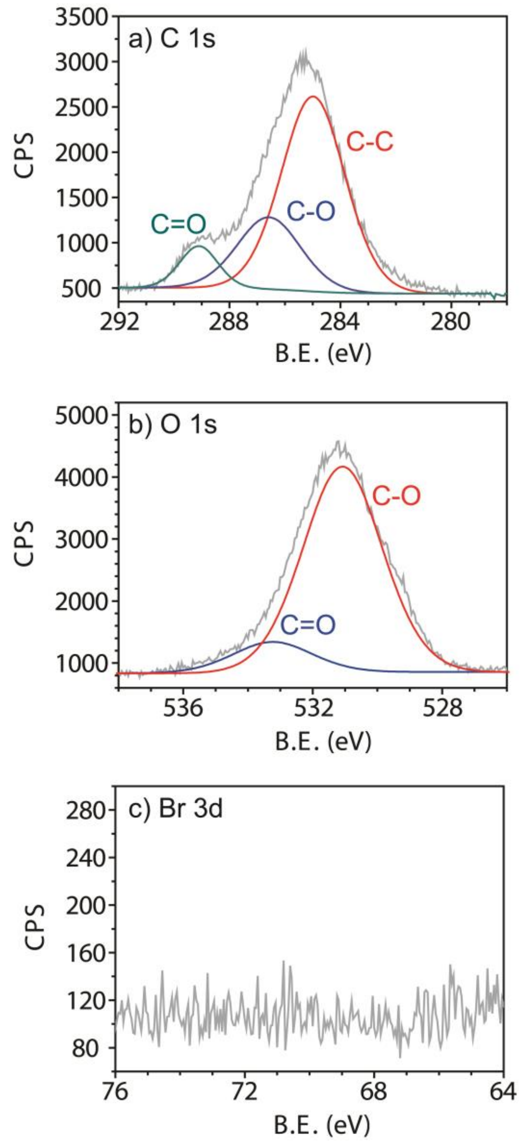

Figure S8. High-resolution XPS a) C1s, b) O1s and c) Br3d spectra of recovered ssPC.

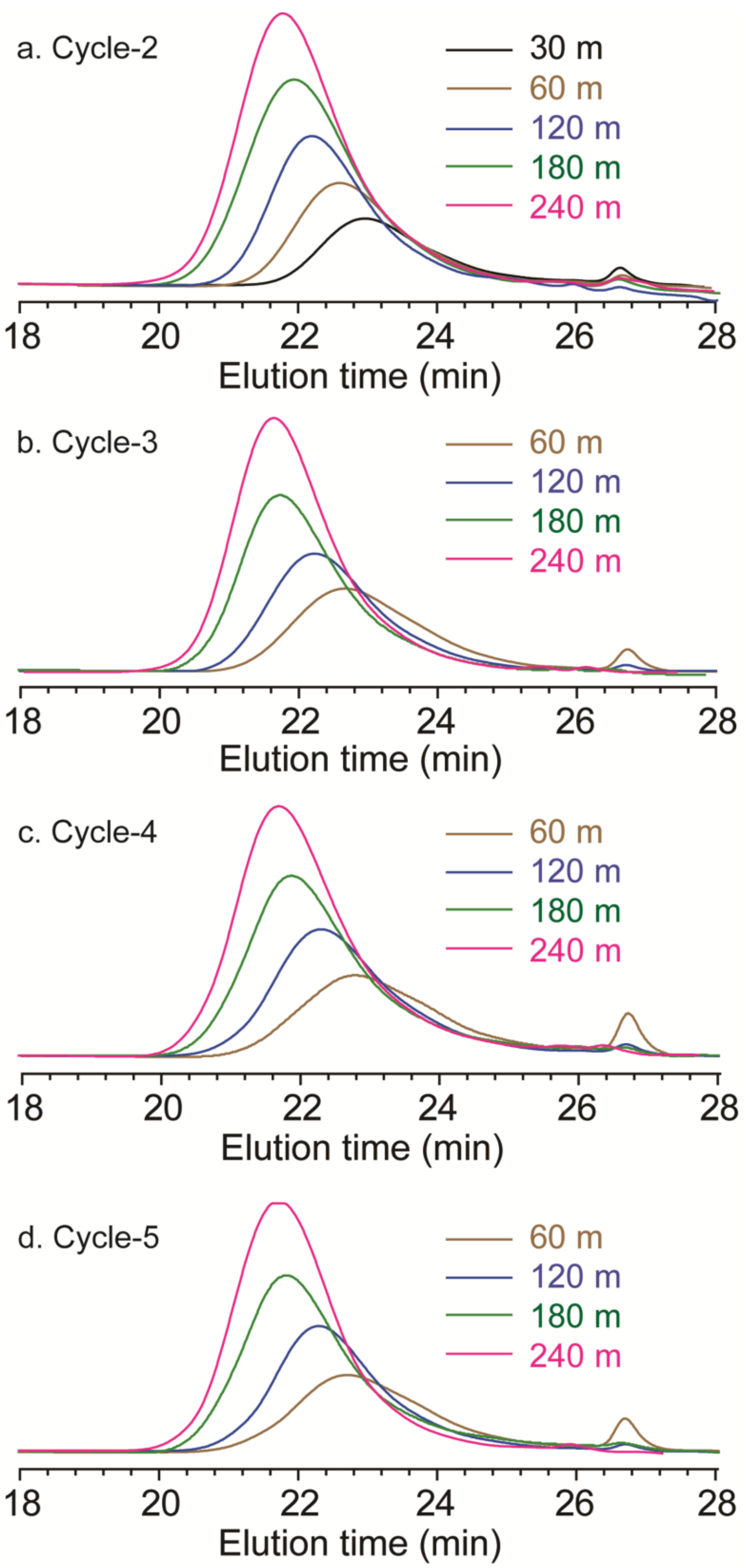

Figure S9. GPC evaluation of PMMAs prepared by the PRP process using recovered ssPC.

Table S3. PRPs of MMA using the recovered ssPC[a].

Entry	Polymerization time (min)	Conv.[b] (%)	$M_{n,th}$[b] (kDa)	$M_{n, GPC}$[c] (kDa)	M_w/M_n[c]
Cycle-2a	30	10.2	1.36	1.53	1.32
Cycle-2b	60	25.7	2.91	3.33	1.29
Cycle-2c	120	60.7	6.42	6.90	1.25
Cycle-2d	180	77.2	8.07	8.52	1.25
Cycle-2e	240	86.3	8.98	9.50	1.23
Cycle-3a	60	26.9	3.04	3.41	1.31
Cycle-3b	120	61.7	6.52	7.11	1.27
Cycle-3c	180	79.0	8.26	8.74	1.25
Cycle-3d	240	86.7	9.03	9.65	1.24
Cycle-4a	60	25.5	2.90	3.32	1.29
Cycle-4b	120	60.7	6.42	6.89	1.25
Cycle-4c	180	80.3	8.39	8.81	1.24
Cycle-4d	240	87.8	9.14	9.72	1.21
Cycle-5a	60	27.9	3.14	3.53	1.30
Cycle-5b	120	61.8	6.53	7.10	1.27
Cycle-5c	180	81.4	8.50	9.02	1.26
Cycle-5d	240	90.4	9.40	9.98	1.25

[a] Supplementary data to Fig. 4. [b] Determined by ^{1}H NMR. [c] Deterrmined by THF GPC.

8. Chain extension

8.1 Preparation of pseudo-diblock copolymer.

Table S4. *In-situ* chain extension experiments for PMMA-*b*-PMMA[a].

Entry[a]	Feed ratio [MMA]:[(macro)-TTC]	Time (h)	MMA Conv.[b] (%)	$M_{n,\,GPC}$[c] (kDa)	M_w/M_n[c]
i.	100	4	> 95	10.5	1.30
ii.	100	4	> 95	20.9	1.27

[a] Supplementary data to Fig. 6a. [b] Determined by ^{1}H NMR. [c] Deterrmined by THF GPC.

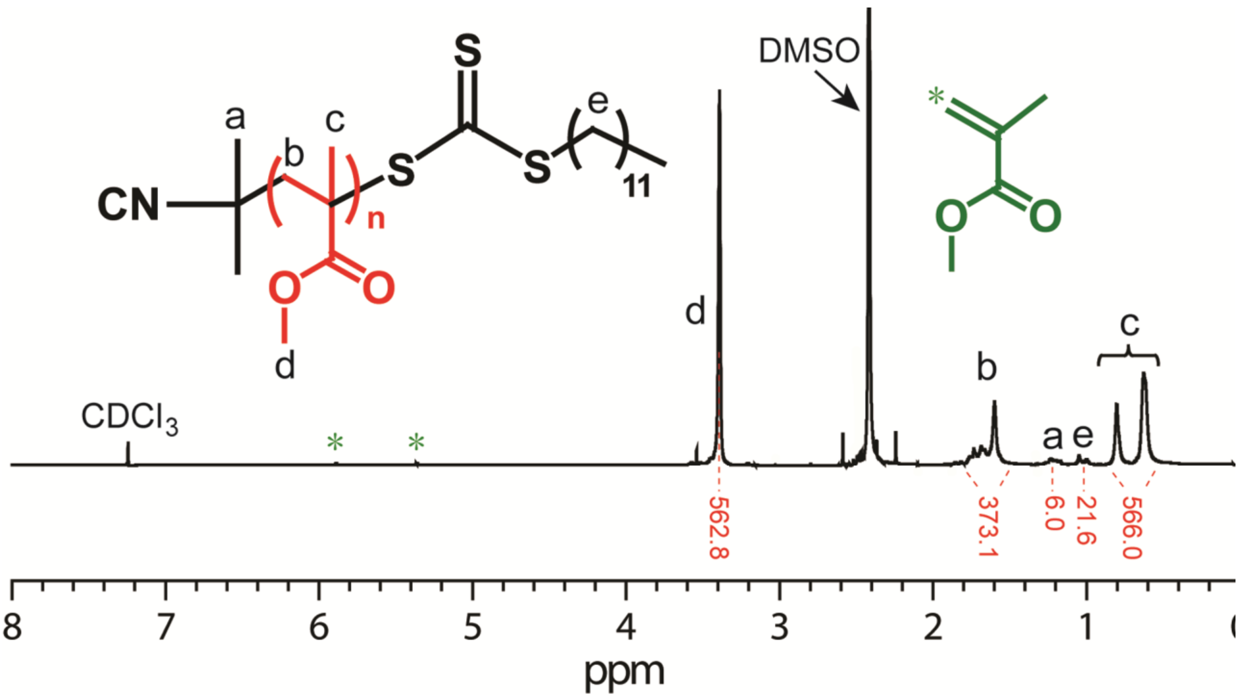

Figure S10. ^{1}H NMR spectrum of crude PMMA-*b*-PMMA using ssPC.

8.2 Preparation of diblock copolymer.

Table S5. Chain extension experiments for PMMA-*b*-PBMA[a].

Entry[a]	Feed ratio [MMA]:[(macro)-TTC]	Time (h)	MMA Conv.[b] (%)	$M_{n,\,GPC}$[c] (kDa)	M_w/M_n[c]
i.	100	3.5	84	9.2	1.30
ii.	100	4	> 92	22.9	1.25

[a] Supplementary data to Fig. 6b. [b] Determined by ^{1}H NMR. [c] Deterrmined by THF GPC.

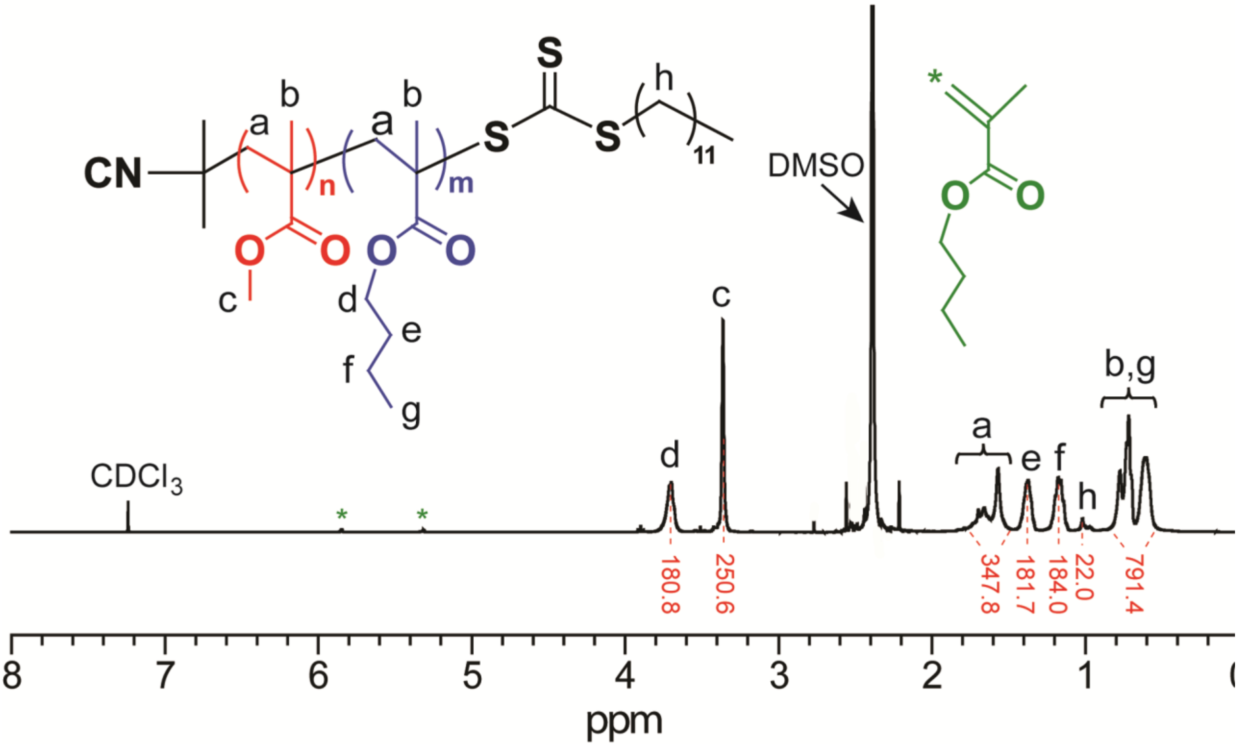

Figure S11. ^{1}H NMR spectrum of crude PMMA-*b*-PBMA using ssPC.

8.3 Preparation of pseudo-triblock copolymer.

General procedure for PMA chain extension was followed. After 16 hours a 1: 2 (v/v) mixture of degassed MA (100 equiv.) used for diblock formation and DMSO was added to the reaction mixture *via* degassed syringe. Samples were taken and conversions were measured using ^{1}H NMR analysis. After another 24 hours, a 1: 4 (v/v) mixture of degassed MA (100 equiv.) and DMSO was added to the reaction mixture *via* degassed syringe for triblock preparation. MA conversion was measured using ^{1}H NMR and the resultant (*pseudo*)triblock copolymer was characterized by GPC measurement.

Table S6. *In situ* chain extension experiments for PMA-*b*-PMA-*b*-PMA[a].

Entry	Feed ratio [MA]:[(macro)CTA]	Polymerization time (h)	MA Conv.[b] (%)	$M_{n,\,GPC}$[c] (kDa)	M_w/M_n[c]
1st block	100	16	> 93	9.2	1.05
2nd block	100	24	> 95	17.3	1.08
3rd block	100	36	> 95	24.5	1.14

[a] Supplementary data to Fig. S12. [b]Determined by ^{1}H NMR. [c]Deterrmined by THF GPC.

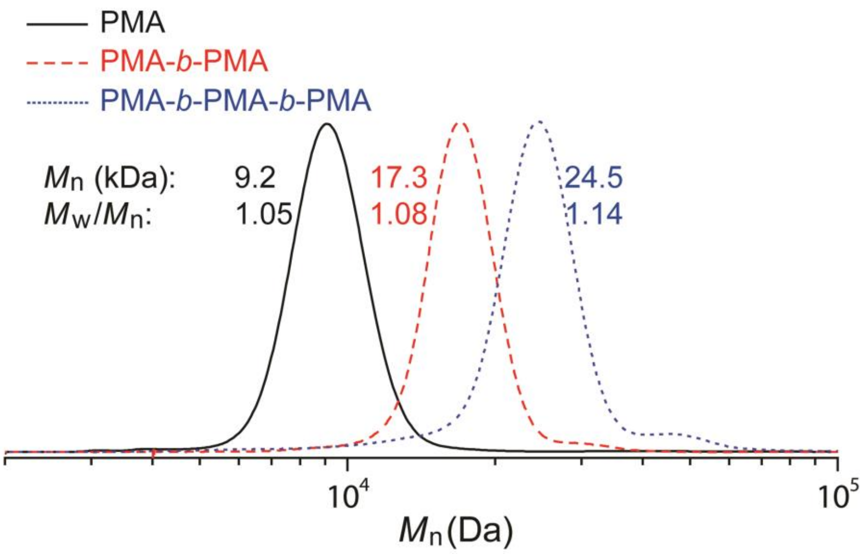

Figure S12. GPC evolution of resultant *pseudo*-triblock copolymer PMA-*b*-PMA-*b*-PMA.

9. CuAAC reactions

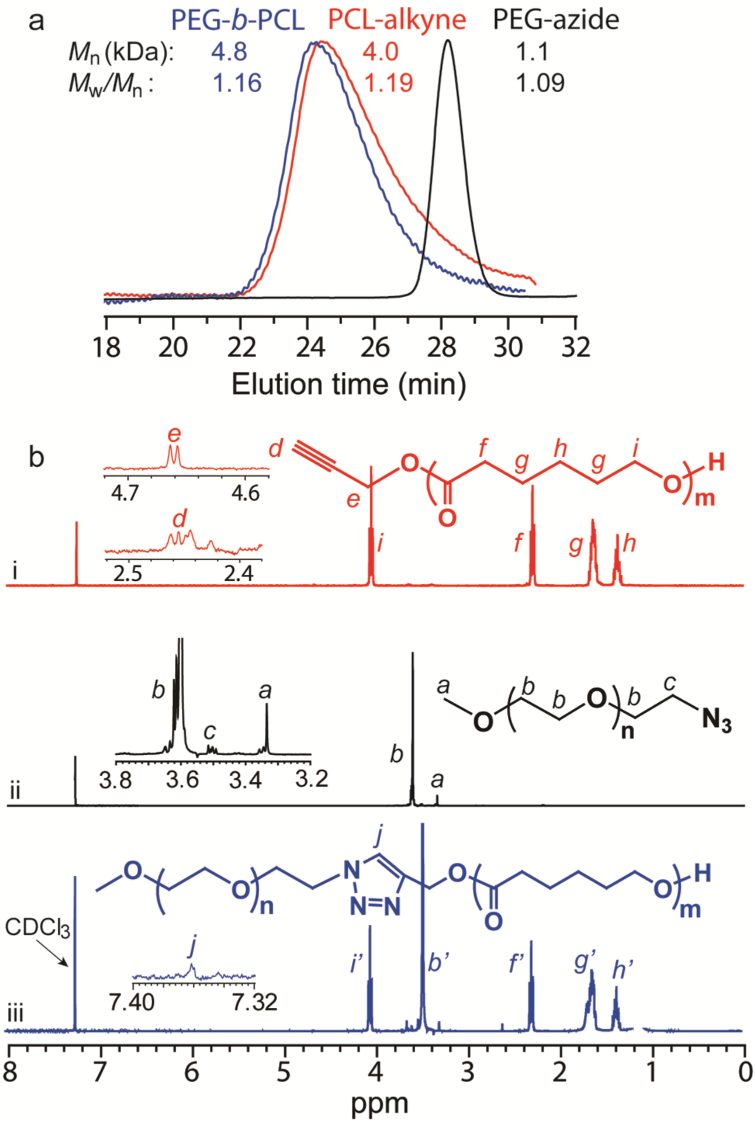

Figure S13. a) GPC evolution of the prepared PEG-*b*-PCL block copolymer *via* 'CuAAC' reaction using ssPC. b) ^{1}H NMR spectra of (i) alkyne-functionalized PCL (PCL-alkyne), (ii) azide-functionalized PEG (PEG-azide) and (iii) the resultant PEG-*b*-PCL block copolymer.

References

1. Ciampolini, M. & Nardi, N. Five-coordinated high-spin complexes of bivalent cobalt, nickel, and copper with tris(2-dime thylaminoe thyl)amine. *Inorg. Chem.* **5**, 41-44 (1966).
2. Tugulu, S., Arnold, A., Sielaff, I., Johnsson, K. & Klok, H.-A. Protein-functionalized polymer brushes. *Biomacromolecules* **6**, 1602-1607 (2005).
3. Lai, J. T., Filla, D. & Shea, R. Functional polymers from novel carboxyl-terminated trithiocarbonates as highly efficient RAFT agents. *Macromolecules* **35**, 6754-6756 (2002).
4. Ren, J. M., Wiltshire, J. T., Blencowe, A. & Qiao, G. G. Synthesis of a star polymer library with a diverse range of highly functionalized macromolecular architectures. *Macromolecules* **44**, 3189-3202 (2011).
5. Fu, Q., Ren, J. M., Tan, S. & Qiao, G. G. Synthesis of novel core cross-linked star-based polyrotaxane end-capped via "CuAAC" click chemistry. *Macromol. Rapid Commun.* **33**, 2109-2114 (2012).
